# Extracellular vesicles from rat-bone-marrow mesenchymal stromal/stem cells improve tendon repair in rat Achilles tendon injury model in dose-dependent manner: A pilot study

**DOI:** 10.1371/journal.pone.0229914

**Published:** 2020-03-12

**Authors:** Clarissa Gissi, Annalisa Radeghieri, Cristina Antonetti Lamorgese Passeri, Marialucia Gallorini, Lucia Calciano, Francesco Oliva, Francesca Veronesi, Andrea Zendrini, Amelia Cataldi, Paolo Bergese, Nicola Maffulli, Anna Concetta Berardi

**Affiliations:** 1 Laboratory of Stem Cells, U.O.C. of Immunohaematology and Transfusion Medicine, Santo Spirito Hospital, Pescara, Italy; 2 Department of Molecular and Translational Medicine and CSGI, University of Brescia, Brescia, Italy; 3 Department of Pharmacy, University G. d’Annunzio, Chieti, Italy; 4 Dipartimento di Sanità Pubblica e Medicina di Comunità, Sezione di Epidemiologia e Statistica Medica, Università di Verona, Verona, Italy; 5 Department of Musculoskeletal Disorders, School of Medicine and Surgery, University of Salerno, Salerno, Italy; 6 Laboratory of Preclinical and Surgical Studies, IRCCS Istituto Ortopedico Rizzoli, Bologna, Italy; 7 Department of Orthopaedics and Traumatology, Azienda Ospedaliera San Giovanni di Dio e Ruggi d'Aragona, University of Salerno School of Medicine, Surgery and Dentistry, Salerno, Italy; 8 School of Pharmacy and Bioengineering, Faculty of Medicine, Keele University, Stoke on Trent, Keele, England, United Kingdom; 9 Centre for Sports and Exercise Medicine, Queen Mary University of London, Barts and the London School of Medicine and Dentistry, Mile End Hospital, London, England, United Kingdom; University of Massachusetts Boston, UNITED STATES

## Abstract

Mesenchymal stromal/stem cells (MSCs) are increasingly employed for tissue regeneration, largely mediated through paracrine actions. Currently, extracellular vesicles (EVs) released by MSCs are major mediators of these paracrine effects. We evaluated whether rat-bone-marrow-MSC-derived EVs (rBMSCs-EVs) can ameliorate tendon injury in an *in vivo* rat model. Pro-collagen1A2 and MMP14 protein are expressed in rBMSC-EVs, and are important factors for extracellular-matrix tendon-remodeling. In addition, we found pro-collagen1A2 in rBMSC-EV surface-membranes by dot blot. *In vitro* on cells isolated from Achilles tendons, utilized as rBMSC -EVs recipient cells, EVs at both low and high doses induce migration of tenocytes; at higher concentration, they induce proliferation and increase expression of Collagen type I in tenocytes. Pretreatment with trypsin abrogate the effect of EVs on cell proliferation and migration, and the expression of collagen I. When either low- or high-dose rBMSCs-EVs were injected into a rat-Achilles tendon injury-model (immediately after damage), at 30 days, rBMSC-EVs were found to have accelerated the remodeling stage of tendon repair in a dose-dependent manner. At histology and histomorphology evaluation, high doses of rBMSCs-EVs produced better restoration of tendon architecture, with optimal tendon-fiber alignment and lower vascularity. Higher EV-concentrations demonstrated greater expression of collagen type I and lower expression of collagen type III. BMSC-EVs hold promise as a novel cell-free modality for the management of tendon injuries.

## Introduction

The incidence of tendon injuries has markedly increased over the past few decades. To date, no viable therapeutic options provide fully successful, long-term solutions; hence, reliable, effective, safe, innovative therapies are required. Recently, cell therapy based approaches have been used to accelerate tendon regeneration and repair.

Tendon function is determined by the biochemical composition and macromolecular structural organization of its extracellular matrix (ECM), which mostly consists of type I collagen with smaller amounts of type III collagen[1] and other components. MMP14 (matrix metalloproteinases 14) is necessary for tendon growth and remodeling during healing[1].

Adult, bone marrow-derived mesenchymal stromal/stem cells (BMSCs), are multipotent stem cells which have been widely studied to treat tissue defects, and are generally considered to be a promising alternative to the current therapeutic approach to tendon injuries[2], although contrasting results have also been obtained. Ectopic ossification, calcification and the higher risk of adhesions formation[3,4], as well as the inherent difficulties in quality control before administration[3,4], are among potential problems when using BMSCs for tendon healing.

Recent investigations suggest that the therapeutic efficacy of MSCs depends on paracrine mechanisms and, more recently, their therapeutic potential has been attributed to the secretion of extracellular vesicles (EVs), which are membrane-enclosed lipid vesicles released by cells as mediators of intercellular communication. Ranging in size from ∼50 nm to > 1μm, EVs carry functional proteins, DNA, mRNA, ncRNA and lipids[5, 6].

Cell-free delivery of bioactive cargos by EV induces the same beneficial responses as stem-cell transplantation, offering remarkable benefits over conventional cell-therapy: for example, EVs avoid the risk of tumorigenesis, and heterotopic ossification and calcification[3,4] and are immunologically unresponsive agents[7, 8]. Finally EVs play a role in tendon-healing by modulating inflammatory responses [9, 10, 11].

This pilot study explores the effect of rBMSC-EVs on an Achilles tendon injury in a rat model to evaluate whether high and low concentrations of EVs derived from rat bone marrow stromal/stem cells without any further supplementation would improve repair of the injured tendon.

## Materials and methods

### Ethics

Sixteen adult male Lewis rats each weighing between 180 and 200 g were bred and maintained in an air-conditioned animal house under specific pathogen-free conditions. All the experiments were conducted according to the protocols of good animal experimentation under the Italian Health Ministry approval n°513/2016-PR and in accordance with international laws and policies (Directive 2010/63/EU of the European Parliament and of the Council, Italian Legislative Decree 26/2014, *Guide for the Care and Use of Laboratory Animals*, NIH publication 85–23, 1985).

### Isolation, culture and characterization of rat BMSCs

**Fig 1:**
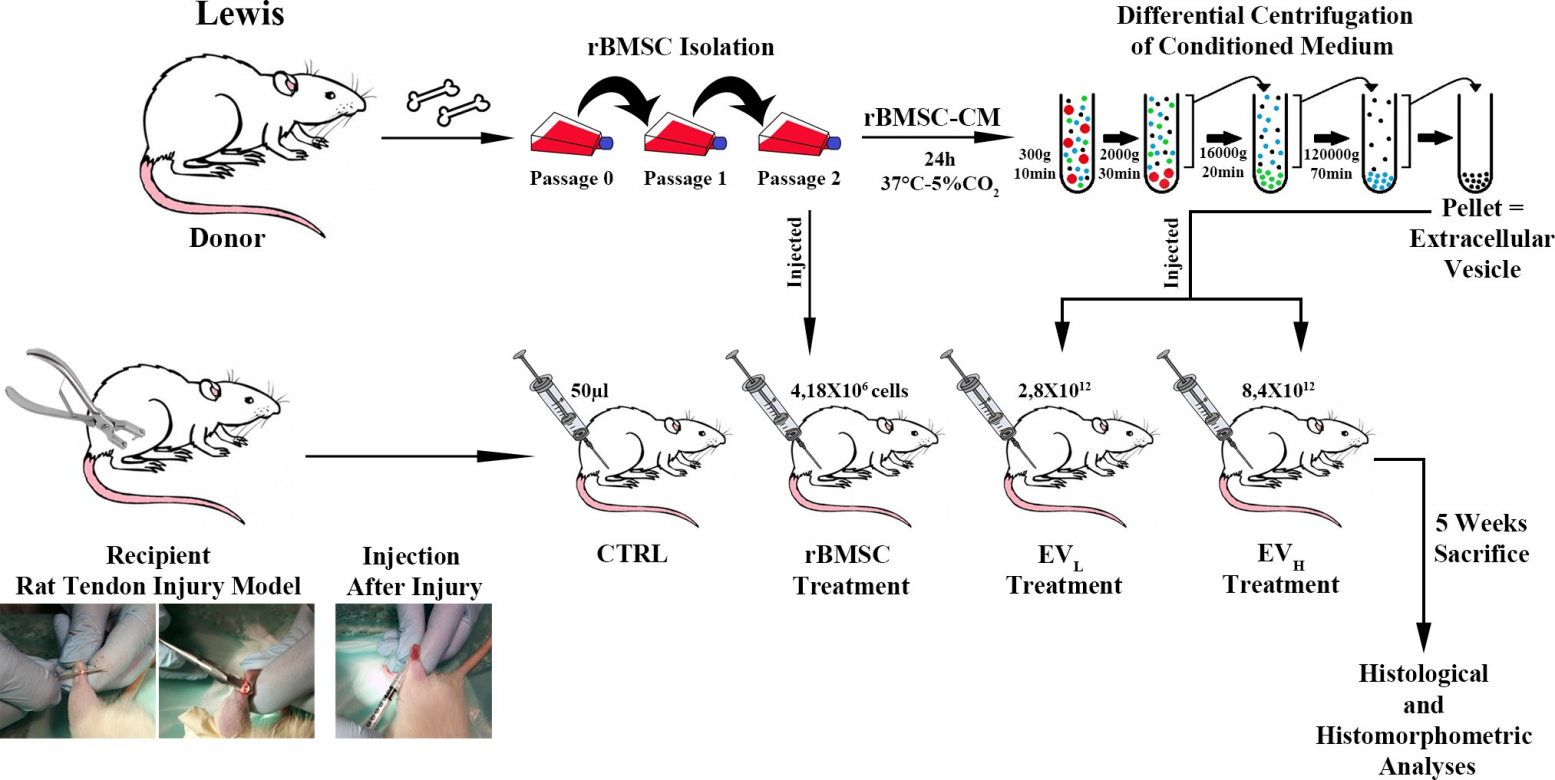
Experimental design. Isolation of rBMSC; EV enrichment by differential ultracentrifugation; Rat Tendon injury model-surgical procedure; Treatment locally micro-injected.

RBMS/SCs were isolated from the tibia and femur bone-marrow from three 6 to 8-week-old male inbred Lewis rats (ENVIGO, Wyton, UK), as recently described [3]. In brief, bone marrow cells at second passage (P2) were used unless otherwise stated. When nearly confluent, the cells in the primary culture were sub-cultured and collected for the study. In addition, MSCs were tested by flow cytometry using specific surface markers, markers following the manufacturer’s instructions, namely mouse monoclonal anti-CD45/FITC, mouse monoclonal anti-CD34/FITC, mouse monoclonal anti-CD90/APC, mouse monoclonal anti-CD44, (all purchased by BD Pharmigen, BD Biosciences, CA, USA) and rabbit monoclonal anti-CD29 (Abcam, Cambridge, UK) counjugated with a goat anti-rabbit IgG fluorescein conjugated secondary antibody (Merck Millipore, Darmstadt, Germany), being negative for CD45 and CD34 and positive for CD44, CD29 and CD90[12,13]. FITC and APC-conjugated nonspecific IgG was used as isotype controls (Biolegend, San Diego, CA, USA). Debris particles were excluded from the analysis by gating on forward and side scatter (FSC and SSC) morphological parameters. At least 10,000 viable and non-debris events in the morphological gate were recorded for each sample. Cells were acquired on a CytoFLEX flow Cytometer (Beckman Coulter, CA, U.S.). The analysis was performed using the CytExpert Software (Beckman Coulter). The stem-cell characteristics of bone-MSCs were routinely confirmed by their trilineage differentiation abilities (adipocytes, osteoblasts, and chondrocytes)[3]. Adipocytes were visualized by Oil Red-O staining, osteoblasts evidenced by Alizarin red, and chondrocytes were visualized with Alcian Blue. Isolated rBMSCs (P0) were cryopreserved according to standard procedures in liquid nitrogen in 90% fetal bovine serum +10% DMSO and stored in liquid nitrogen in vials until transplantation (Fig 1).

### EV isolation

The protocols used to separate EVs were in accordance with the most recent guidelines (MISEV 2018) [14]. FBS and bovine serum albumin (BSA) solution (Life Technologies) used for EV production were depleted from endogenous EVs prior to use by ultracentrifugation at 120,000 g for 5 hours, using an Ultracentrifuge Optima L-100K Ultracentrifuge (Beckmann Coulter). After centrifugation, the FBS and BSA supernatants were filtered with a 0.22 μm filter (ThermoFisher Scientific, *Inc*.,Rochester, NY, USA) and stored in aliquots at -80°C.

rBMSC 80%-confluent cell plates (1–2 ×106 cells/mL) were washed 2 times with PBS without calcium and magnesium, then incubated for 24 hours in DMEM/F12 (1:1) (Life Technologies, Carlsbad, CA, USA) supplemented with 1% EV-free FBS and 0.25% EV-free BSA at 37°C and 5% CO_2_. Cell viability was assessed using Trypan blue exclusion methods. Presence of apoptotic cells was checked by flow-cytometric assay for annexin V expression (data not shown). EVs were prepared from the cell-culture media using differential centrifugation steps as previously described with some modification[15]. All preparation and centrifugation steps were performed at 4°C. Briefly, collected cell-media were subjected to a first centrifugation at 300×g for 10 min to remove non-attached cells, followed by a second centrifugation at 2,000×g for 30 min to remove apoptotic bodies, and finally a third centrifugation at 16,000×g for 20 min to remove residual ABs and cell organelles. EVs were then pelleted from the purified supernatant by centrifugation at 120,000×g for 70 min in 38 mL polycarbonate tubes (Beckman #355631). This EV-enriched pellet was resuspended in PBS, and the centrifugation was repeated again as above. The final EV pellets was thoroughly drained, rapidly frozen in liquid nitrogen and stored at -80°C until their use.

### Atomic Force Microscopy (AFM) imaging

For AFM, EVs were diluted 1:10 in MilliQ water and 5 μL of preparation were spotted on to freshly-cleaved mica substrates (Agar Scientific, Stansted, Essex CM24 8GF United Kingdom). Each substrate was dried at 30°C for 10 minutes and analyzed with a NaioAFM (NanoSurf), equipped with Multi GD-G probes (BudgetSensors). Measurements were performed in dynamic mode, and scanning parameters were set according to the probe manufacturer. AFM images were processed using WSxM 5.0 (http://www.wsxmsolutions.com). EV size distribution was performed on ImageJ, analyzing 10 different AFM pictures. A total of 550 particles were measured. Only particles with a diameter of at least 30 nm were analyzed; particle roundness threshold was fixed to 0.800, in order to eliminate possible artifacts deriving from non-EV objects. EV weighted mean, median and mode diameters were calculated using GraphPad Prism 6, which was also used to plot EV size distribution data[16].

### Colloidal nanoplasmonic assay

EV preparations were checked for purity and titrated by adapting the colorimetric nanoplasmonic assay we previously developed [17, 18]. The assay synergistically exploits nanoplasmonics of gold nanoparticles (AuNPs), AuNP-protein corona and AuNP membrane collective interactions. Briefly, citrate capped spherical AuNPs adsorb and cluster at the EV membrane in pure formulations. Instead, if the formulation contains exogenous protein contaminants, AuNPs are preferentially coated by those contaminants, forming a AuNP-contaminant corona which keeps AuNPs dispersed and prevents them from clustering to the EV membrane. Clustering drives a red shift of the AuNPs localized surface plasmon resonance (LSPR) absorption peak, which in turn is directly related to the purity grade of the formulation. Isolated EV samples were resuspended in sterile milliQ H2O (Q GRAD2 Millipore) and mixed with a final concentration of 3nM, 15 nm AuNPs. The blue shift of the different AuNP-EV solutions was quantified by collecting the spectra with a microplate UV–vis reader (EnSight Multimode Reader, Perkin- Elmer), acquired with 1 nm step size in a wavelength window ranging from 400 nm to 900 nm. The AuNPs aggregation index (AI) was defined as

$$AI=\frac{{\mathrm{Absorbance}}_{524\;\mathrm{nm}}}{{\mathrm{Absorbance}}_{650\;\mathrm{nm}}+{\mathrm{Absorbance}}_{900\;\mathrm{nm}}}$$

For quantification of EV number, a calibration curve was built by using Synthetic phosphatidylcholine (PCh) liposomes at different concentrations, prepared as previously described[16]. Briefly, the proper amount of POPC (1-palmitoyl–2- oleoyl-sn-glycero-3-phosphocholine, Avanti Lipids) was dissolved in chloroform/methanol 6:1 (v/v). A stream of nitrogen and overnight vacuum drying were applied to obtain a lipid film by evaporating the solvent. The film was swollen and re-suspended in warm (50°C) 0.9% NaCl solution by vigorous vortex mixing. To prepare liposomes with narrow distribution, the dispersion was tip-sonicated for 30 minutes.

### EV biophysical characterization

EV preparation purity, morphology and size distribution were investigated through Atomic Force Microscopy and Colloidal Nanoplasmonic assay (CONAN). The latter exploits the properties of colloidal solutions containing gold nanoparticles (AuNPs) and EVs. All relevant data of our experiments were submitted to the EV-TRACK knowledgebase (EV-TRACK ID: EV180057).

### Protein preparation from cells and EVs

Proteins were isolated from rBMSCs and from EVs derived from the same rBMSCs using M-PER Mammalian protein extraction reagent supplemented with 1% of Halt Protease, EDTA-free phosphatase-inhibitor cocktail and 1% of EDTA solution (all from Thermo Fisher Scientific), according to the manufacturer’s indications[6]. Protein extracts were stored at -20°C. The concentration of total protein preparations was quantified by BCA assay (QuantiPro^TM^ BCA assay kit for 0.5–30 μg/mL protein, Sigma-Aldrich, Saint Louis, MO, USA).

### Western blotting

Equivalent amounts of cells and EV protein lysates were electrophoresed and transferred to nitrocellulose membranes. Nitrocellulose membranes were then blocked in 5% non-fat milk or 5% BSA, 10 mmol/l Tris-HCl pH 7.5, 100 mmol/l NaCl, 0.1% Tween-20, and probed with the following primary antibodies: mouse monoclonal anti-GM130 (1:1000, BD Biosciences, 610822), mouse monoclonal anti-Annexin V (1:500, Santa Cruz Biotechnology, Dallas, U.S., sc-74438), mouse monoclonal anti-Annexin XI (1:500, Genetex, CA, U.S., GTX33010), mouse monoclonal anti-TSG101 (1:500, Santa Cruz Biotechnology, sc-7964) rabbit monoclonal anti-TERT (1:500 1000 Rockland Immunochemicals Inc., PA, U.S., 600-401-252S), mouse monoclonal anti-Pro-COL1A2 (D-6) (1:200, Santa Cruz Biotechnology, sc-166572), rabbit monoclonal anti-Integrin beta1 (1:2000, ab179471), rabbit monoclonal anti-MMP3 (1:1000, ab52915), and rabbit monoclonal anti-MMP14 (1:2000, ab51074) (all purchased by Abcam) and incubated in the presence of specific, HRP-conjugated secondary antibodies. Immunoreactive bands were visualized using ECL detection system (Amersham International, L.C., UK) and by ChemiDoc XRS Imaging system (BioRad, CA, U.S.) or G:Box Chemi XT Imaging system (Syngene, Cambridge, UK).

### Tendon cell cultures

Rat tendon cells were isolated according to previously described protocols [19, 20, 3]. The Achilles tendons were harvested from three male, 6 to 8-week-old, inbred Lewis rats; the animals were anesthetized and then euthanized using CO_2_ in specially designed chambers. Cells were isolated from tissue sample by washing several times with Dulbecco’s phosphate-buffered saline (PBS) without Ca2+ and Mg2+ and supplemented with 1% penicillin/streptomycin (Invitrogen, Life Technologies,). Small pieces of fresh isolated tendon were carefully dissected and mechanically disaggregated with the aid of fine watchmaker forceps to maximize the interface between tissue and medium. Finally, the tendons were immediately placed on Petri dishes 60 mm in diameter (Greiner CELLSTAR dish, Sigma-Aldrich), containing 5 mL of α-MEM supplemented with 20% heat-inactivated foetal calf serum (FCS), 1% L-glutamine and 1% penicillin/streptomycin (Gibco, Invitrogen, Life Technologies) at 37°C in 5% CO2 and air with a refresh of medium every 2–3 days. Tenocytes were harvested with StemProAccutase (Life technologies) and centrifuged at 1,500 rpm for 5 min when cells migrated out of tendon pieces and reached 60–80% confluence (day 19). Collected tenocytes were immediately cultured to avoid phenotype drift with further in vitro passages. Cells from P2 were used for all experiments, and the medium was changed every three days.

### MTT assay for cell proliferation

Proliferation assays were performed in 96-well plates at a density of 2x10^3^ tenocyte rat cells/well and assayed at 48 hr. In brief, tenocytes from rats were cultured for 48 hr. in alpha-MEM with 10% FBS. Then, the medium was replaced with fresh medium alone (used as a control) or with EVs (2,8x10^12^ or 8,4x10^12^ /mL). At established time points, the medium was replaced with a fresh one containing 0.5 mg/mL MTT (3‒[4,5‒dimethyl‒thiazol‒2‒yl‒]‒2,5‒diphenyl tetrazolium bromide) growth assay (Sigma-Aldrich), and the cells were incubated for 4 hr at 37 C. After a further incubation of the samples in DMSO for 30 min at 37 C, 200 μL of each medium were transferred into a 96-well plate, and the absorbance at 570 nm was measured using a Multiscan GO microplate spectrophotometer (Thermo Fisher Scientific). The values obtained in the absence of cells were considered as background and subtracted from the optical density values of the samples. Three independent experiments were performed under the same experimental conditions. In select experiments, rBMSC-EVs were incubated with trypsin (1 mg/mL, Sigma) for 1 hr at 37°C and re-isolated by ultracentrifugation prior to application as previously described [21].

### Cell migration

Cell migration was performed by scratch assays. Rat tenocytes were grown to confluence in a 96-well plate and the scratch was made in the cells using a P10 pipette tip. After a PBS wash, cultures were supplemented with or without rBMSC-EVs in reduced FBS conditions (1% FBS). Cell migration into the empty area was monitored by bright field microscopy (NIKON, NY, U.S.) at 0 or 24 hr. The reduction in the empty area was determined using the ImageJ software (Version 1.49 v, RRID:SCR_003070; NIH, Bethesda, MD). The data were statistically analyzed using GraphPad Prism 5 software (GraphPad Software, Inc.). All experiments were repeated ≥3 times. In select experiments, rBMSC-EVs were incubated with trypsin (1 mg/mL, Sigma) for 1 hr at 37°C and re-isolated by ultracentrifugation prior to application as described above.

### Immunofluorescence staining

The tendon-derived cells were seeded with 5×10^3^ vital cells per well in 8-well chamber slides (Thermo Fisher Scientific), in triplicates with or without rBMSC–EVs as described above. After 0, and 7 d of culture the tendon-derived cells were fixed with pure acetone for 10 min at −20°C. Then, washed for a few minutes with PBS. Cells were incubated for 30 min at room temperature with PBS containing 5% Bovine Serum Albumin (BSA) (Kedrion Group S.P.A., Lucca, Italy) for protein blockage. Primary antibodies for Anti-type I (1:2000) and Anti-type III collagen molecules (1:500) (Sigma-Aldrich) and secondary antibodies fluorochrome were diluted in PBS containing 5% BSA. Cells were incubated for 1h at 37°C with primary antibodies, and for 1 h with the appropriate secondary antibody fluorochrome at 37°C and then washed a few times with PBS containing 5% BSA. Molecule staining Alexa Fluor 488 (Life Tecnologies) was used for type I collagen and Alexa Fluor 568 (Life Tecnologies) was used for type III collagen. Finally, a solution of 4′,6-diamidino-2-phenylindole (DAPI; Sigma-Aldrich) diluted 1000 times with PBS was added, allowed to stand at room temperature for 5 min and washed three times with deionised water. For image analysis all digital images were captured with NIS-Elements Imaging Software (Nikon Instruments INC.). Mean fluorescence intensity (MFI) of the negative control was subtracted from that of each sample. The MFI (mean intensity of Anti-type I-Alexa fluor 488 and Anti-type III-PE collagen molecules) fold change was obtained by normalising MFIs (mean intensity of Anti-type I-Alexa Fluor 488 and Anti-type III-PE collagen molecules) to the related untreated cell fluorescence. [22]. In selected experiments, rBMSC-EVs were incubated with trypsin (1 mg/mL, Sigma) for 1 hr at 37°C and re-isolated by ultracentrifugation prior to application as described above.

### Animal surgery and pilot study design

Sixteen 6–8 week-old male inbred Lewis rats (ENVIGO) were housed under controlled conditions in the Interdepartmental Service Centre—Station for Animal Technology, University of Rome “Tor Vergata” (Italy), supplied with standard diets (4RF18; Mucedola srl, Italy) and water *ad libitum*. At time of surgery, general anesthesia was induced with an intramuscular injection of tiletamine/zolazepam (50 mg/kg) (Zoletil 100) and xylazine (10 mg/kg) (Rompun Bayer). Under general anesthesia, the right Achilles tendon was laid open by a skin incision of approximately 10 mm on the medial side of the right hind-limb of each rat. In each animal, a bilateral Achilles tendon defect 2 mm in diameter was produced as described in our recent study[3].

The animals were divided into four groups (4rats /group). The injured tendon was filled (immediately after skin suture) with 50 μL of Phosphate-buffered saline (PBS), which is a physiological buffer solution containing: (1) PBS alone (control group: CTRL); (2) “rBMSCgroup”: 4 x10^6^ cells; (3) “EV_L_ group”: 2.8x10^12^ EVs; (4) “EV_H_ group”: 8.4x10^12^ EVs. (Fig 1) Post-operatively, antibiotics and analgesics were administered: 0.5 mL/kg baytril (Bayer AG) and 0.1 mL/kg/day rimadyl (Veterfarma SpA). After 30 days, the animals were anesthetized and then euthanized using CO_2_ in specially designed chambers. Both tendons were explanted, embedded in optimal cutting temperature (O.C.T.) compound medium (Sakura Finetek USA), and quickly frozen in liquid nitrogen-cooled isopentane for sectioning at a thickness of 12 μm on a Leica cryostat and then stained with hematoxylin and eosin (H&E), or Picro-Sirius red solution for histological and histomorphometric analyses. (Fig 1)

### Histological and histomorphometric examination

Three consecutive slides stained with H&E were evaluated through a modified semi-quantitative score obtained using Soslowsky, Svensson, and Cook’s scoring systems (Table 1)[3]. These scores take into account 4 different parameters: fiber structure, cellularity, vascularity and cartilage formation, rating each from 0 to 3. The overall score is the sum of the values of each parameter and, as indicated in Table 1, the best possible score is 0; the higher the values are, the worse the pathology.

**Table 1:** Semiquantitative histomorphometric scoring system as modified by Svensson, Soslowsky, and Cook.

Parameter	Score (points) 0 (Health Tendon)	Score (points) 1	Score (points) 2	Score (points) 3
***Fiber Structure*, *Svensson and Soslowsky Modified***	Normal parallel collagen fibers	Mild changes (collagen fibers <25% disorganized, increased waviness)	Moderate changes (collagen fibers 25–50% disorganized, deterioration of fibres)	Marked changes (collagen fibers >50% disorganized, hyalinisation)
***Cellularity (Aspect)*, *Cook Modified***	Elongated spindle shaped nuclei with no cytoplasm at light microscopy	Nucleus becomes more ovoid without conspicuous cytoplasm	Increased roundness and size of the nucleus and little cytoplasm	Round nucleus and with abundant cytoplasm
***Vascularity*, *Svensson Modified***	Few vessels, parallel to collagen fiber	Slight increase of vessels	Moderate increase of vessels	Markedly increased of vessels
***Cartilage Formation***	No cartilage	Isolated cartilage nodules	Moderate cartilage formation (25–50%)	Extensive cartilage formation (>50%)

Histological images were analyzed using an Aperio ScanScope digital image analysis system. The entire tendon surface was analyzed, and histological scores were assigned by two blinded independent histologists.

Three other consecutive slides for each sample were stained with Picro-Sirius red solution. Using an optical microscope (BX51, Olympus Italia Srl) at 8X magnification, collagen type I and type III percentages were calculated by automated measurement of the red-orange fiber area (collagen type I) and pale green fiber area (collagen type III) from these slides[3].

### Statistical analyses

*In vitro* data are typical results from a minimum of three replicated independent experiments, and are expressed as mean ± SD. Comparison of individual treatment was made using Student’s *t* test. A one-way ANOVA test was used for comparison of three or more groups, and was followed by Tukey’s *post hoc* test. Differences were considered significant when * *p* < 0.05, * **p < 0.01, *** *p*< 0.001, **** *p* < 0.00001.

According to the “*One-between*, *one within factor*, *repeated measures ANOVA*” formula based on the ‘*resource equation*’ approach[23], the minimum and maximum numbers of rats required in our study were 2.25 (rounded up to 3) and 3.5 (rounded up to 4) rats/group-treatment, respectively.

Ordered probity regression models and linear regression models with the treatment-group as covariate, followed by Bonferroni-adjusted *post hoc* test, were used to compare the effect of treatments on the histological scores and the collagen ratios, respectively. Cluster robust standard errors were computed in order to take the correlation among the two histologists per rats into account. Statistical analysis was performed using the STATA v.14.2 software.

Statistical analyses were performed using the GraphPad Prism Software 5.0. Data are expressed as mean values ± standard deviation (SD). Statistical significance was set at *p* ≤ 0.05.

## Results and discussion

**Fig 2:**
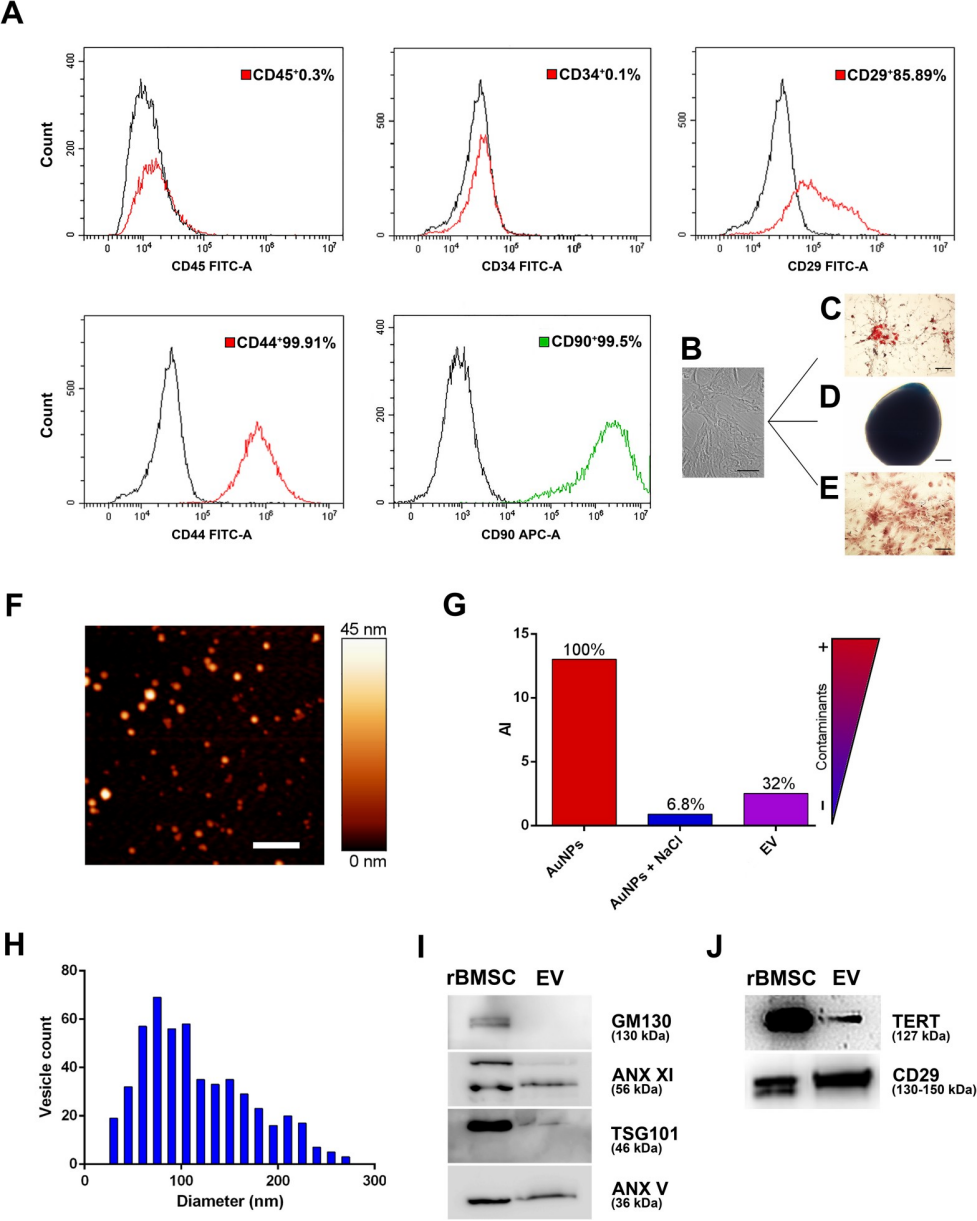
rBMSC characterization. An example of rBMSC phenotype and functional analysis. **(A)** Flow cytometry analysis of MSCs was performed with FITC-conjugated antibodies (red histograms) and PE-conjugated antibodies (green histogram) to detect cell surface markers. The expression of isotype controls is shown in the black histograms. Flow cytometry analysis revealed typical CD44, CD29 and CD90 mesenchymal surface-marker expression, without CD45 and CD34 hematopoietic surface markers, and confirmed that only mesenchymal cells had been isolated (CD45^-^CD34^-^CD29^+^CD44^+^CD90^+^ cell population). **(B, C, D, E)** *In vitro* differentiation of Mesenchymal Cells. The cells were shown to be multipotent stem-cells by differentiation capacity along osteogenic, chondrogenic and adipogenic lineages.**(B)**: Cells were incubated in lineage-specific induction media and then analyzed by histochemical and cytological staining for **(C)** Adipocytes (Oil Red-O staining), **(D)** Chondrocytes (Alcian blue staining) and **(E)** Osteoblasts (Alizarin Red staining). *Characterization of rat BMSC derived extracellular vesicles*. **(F)** AFM topography image of the EV preparations. Scale bars are 1 μm. **(G)** CONAN assay. **(H)** Size distribution and diameter of EV samples. **(I)** Representative western blots showing the expression of: GM130, ANX XI, TSG 101, ANX V, TERT and CD29 (Integrin beta1) in rBMSC and rBMSC-derived EVs. Uncropped gel from different gels. Experiments were performed 3 times with similar results.

We separated EVs from rBMSC-conditioned media using a previously published, validated protocol[3], and characterized them according to the most recent guidelines of the International Society for Extracellular Vesicles (MISEV 2018)[14] (Fig 2A, 2B, 2C, 2D and 2E).

Biophysical analysis data indicate that enriched EV populations were obtained from rBMSCs. The purity, size distribution and number of separated EVs were revealed by Atomic Force Microscopy (AFM) and Colloidal nanoplasmonic (CONAN) assay (Fig 2F, 2G and 2H). Western blot analysis, using antibodies against surface and internal makers, excluded contamination by other membrane fragments (GM-130), confirmed EV-specific marker-expression (Annexin XI, Annexin V and TSG-101), TERT-expression (in accordance with a recently-discovered biomarker on amniocyte-derived EVs[24]) and characteristic CD29 protein-expression of the parental MSCs (Fig 2I).

### rBMSCs-EV express Pro-collagen1A2 and MMP14

**Fig 3:**
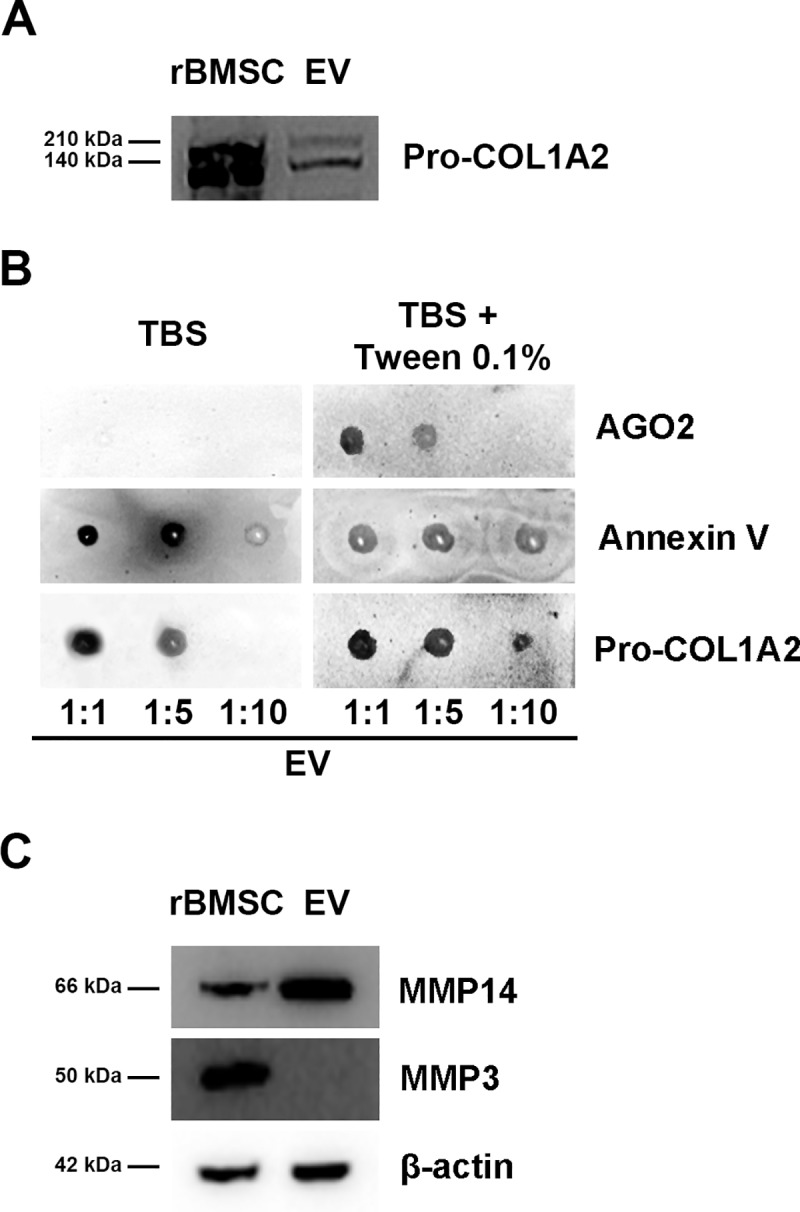
**(A)** Representative Western blots showing Pro-collagen1A2 expression in rBMSC and rBMSC-derived EVs. Panel A, representative Western blotting of Pro-COL1A2. Uncropped gel**. (B)** *Demonstration that Pro-COL1A2 labels the surface membrane of EVs*. EVs were dot blotted on nitrocellulose membrane in a dose range followed by immunoprobing with anti-Pro-COL1A2 (1:200) or anti-Annexin V (1:500) (Santa Cruz Biotechnology) or anti-Ago2 (1:1000) (OriGene Technologies), respectively, and horseradish peroxidase (HRP)-conjugated secondary antibodies in the presence or absence of detergent [0.1% (v/v) Tween20] for chemiluminescence detection. After immunoreaction with the specific antibody, dot blot showed the protein is mainly associated to EV surface rather than in EV lumen; indeed, Pro-collagen1A2 was detected even in absence of Tween-20, which permeabilizes EV membranes and allows the detection of EV lumen-associated proteins. AGO2 and annexin V are here used as controls: the first as an indicator of EV integrity the latter as representative of EV membrane-bound protein. **(C)** *Matrix metalloproteinase 14 and Matrix metalloproteinase 3 detection by representative Western blotting*. Uncropped gel. Matrix metalloproteinase 14 was highly concentrated in EVs secreted by rat bone marrow stem/stromal cells. Matrix metalloproteinase 3 was present only in rBMSCs.

As tendon ECM is largely composed of Collagen Type I, we investigated Collagen Type I precursor-expression in EVs by western blot. We identified the presence of Pro-collagen1A2 in EVs, and demonstrated, by dot blot, that Pro-collagen1A2 predominantly labels the EV-membrane surface (Fig 3A and 3B).

WB analysis confirms the presence of MMP14 (also known as membrane type 1 MMP, or MT1-MMP) in rBMSC-EVs (Fig 3C) in accordance with other studies. Gulotta et al. have hypothesized that MMP-14 in tenocytes may induce regeneration[1]. Furthermore, MMP14 in EVs are crucial in increasing cell migration and activating pro-MMP2, as well as in the degradation of ECM-proteins [25]. We were further interested to analyze MMP3, which seems to be crucial in connective-tissue remodeling and has been found in adipocyte-derived EVs [26]. Furthermore, Wang et al. have been shown that EV derived from tendon stem cells, injected on rat tendon injury model significantly decreased MMP3 expression and increased the expression of Collagen type I promoting tendon healing [27, 25]. However, our WB analysis showed the complete absence of MMP3 in rBMSCs-EVs, despite its confirmed presence in the originating rBMSCs (Fig 3C).

### rBMSC–EVs induce proliferation, migration and expression of collagen type I of rat tendon-derived cells

**Fig 4:**
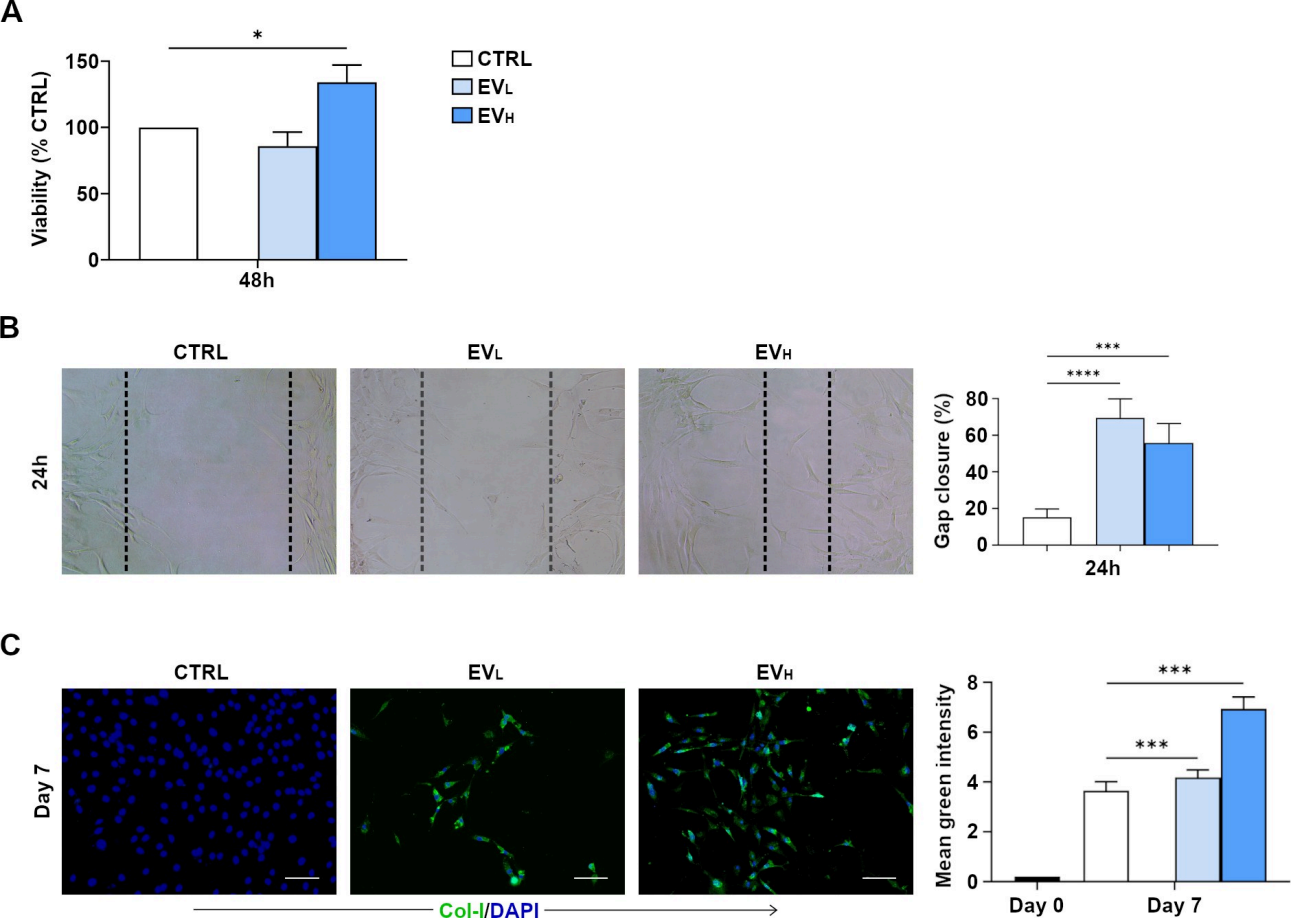
rBMSC-EVs promote tendon-derived cell proliferation and migration, and increase expression of collagen type I. **A)** Effects of rBMC–EVs on cell proliferation in cells isolated from rat tendons. Different concentrations of rBMC–EVs (2,8x10^12^ or 8,4x10^12^ /mL) for 48 hr determined by MTT assay. Data are reported as means ± SD. All experiments were repeated 3 times. **B)** tenocyte migration assessed by scratch wound healing assay at 24 hr with or without rBMSC -EVs (2,8x10^12^ or 8,4x10^12^ /ml). Representative 100x images with percentage gap closure are shown. **C**) Collagen type I, expression of tendon-derived cells in an *in vitro* culture isolated from four male 6 to 8-week-old inbred Lewis rats, and stained as described in material and methods section under Immunofluorescence Staining. Representative images from 3 independent experiments. Expression of collagen type I after 7 d of culture in green fluorescence and in blue fluorescent (nucleus staining) images showing the tendon-derived cells. Quantification of the immunohistochemistry in rBMC–EV_H_ induced the expression of collagen type I significantly after 7 d of in vitro culture. The mean fluorescent intensity/pixel was measured and expressed to corresponding tendon-derived cell. Collagen type I Intensity (Total Area was quantified by anti-collagen type I) was measured by Nikon software. Data are expressed as mean ± SD for 3 independent experiments for samples run in triplicate. Scale bar (a.): 50 μm. Data shown as mean ± SD, and represent triplicate experimental replicates. White scale bar: 20mm. Black scale bar: 100 nm. *p<0.05; **p<0.01; ***p<0.001; **** *p* < 0.00001.

First, interaction of rBMC -EVs on the recipient tendon-derived rat cells was assessed. We analyzed the metabolic activity of tenocytes when exposed to different concentrations of rBMC -EVs (2,8x10^12^ or 8,4x10^12^ /ml) for 48 hr. The MTT results showed that rBMC -EV treatment with the higher concentration (rBMC-EV_H (High)_) exerted a significant effect on tenocyte proliferation, metabolic activity being increased by up to 34%. (Fig 4A). Scratch wound healing assays showed that both the lowest and highest rBMC–EVs induced a significant pro-migration effect in the tenocytes (Fig 4B). Next, we determined and measured the type of collagen deposited by tendon-derived cells after stimulation with rBMC -EVs. Collagen accumulation was evaluated by immunofluorescent staining of cells cultured on chamber slides for 7 days. The expression of collagen type I in the presence of rBMC-EVs increased in a dose-dependent manner. Furthermore, in the presence of rBMC–EV_H_ the expression of collagen type I was significantly higher compared to both untreated cells (used as a control) and rBMC–EV_L_ (Low) (Fig 4C). Collagen type III was not found to be present in any culture conditions (data not shown). These data demonstrate that all BMSC -EVs induce proliferation and migration of tendon-derived cells and increase their collagen type I expression. From the data obtained, we can hypothesize that the combination of all these effects may positively regulate Achilles tendon repair. Higher doses of rBMSC -EVs induced proliferation and expression of collagen type I, while both lower and higher doses of rBMSC–EVs induced migration. As it has been previously suggested for other tissue models [21], EVs may be assumed to play an important role at Achilles tendon injury sites in vivo and where, in all probability, an EV-concentration gradient effect exists. With both lower and higher concentrations of EVs, tenocytes may be recruited at a distance, and tenocytes can increase in number at higher EV concentrations and increase collagen type I fiber formation.

Recent research has already demonstrated that surface-associated proteins are key to EV-bioactivity [21]. Thus, we verified in our model whether the bioactivity effects of EVs were dependent on proteins bound to the EV-membrane. EV membrane–bound proteins were digested through pre-treatment with trypsin. In agreement with Jiajia Xu et al. [21], our data showed that pre-treatment with trypsin completely abrogated the proliferation, migration and expression of collagen type I effect of rBMC–EVs.(S1A and S1B Fig and S1C Fig respectively). Our results showed that rBMC—EV uptake and bioactive effects were dependent on membrane-bound protein interaction with recipient tendon cells. Thus, these results can let us speculate that less targeting of EVs can reduce their effects versus tendon repair.

### rBMSC-EVs promote Achilles tendon injury repair *in vivo*

**Fig 5:**
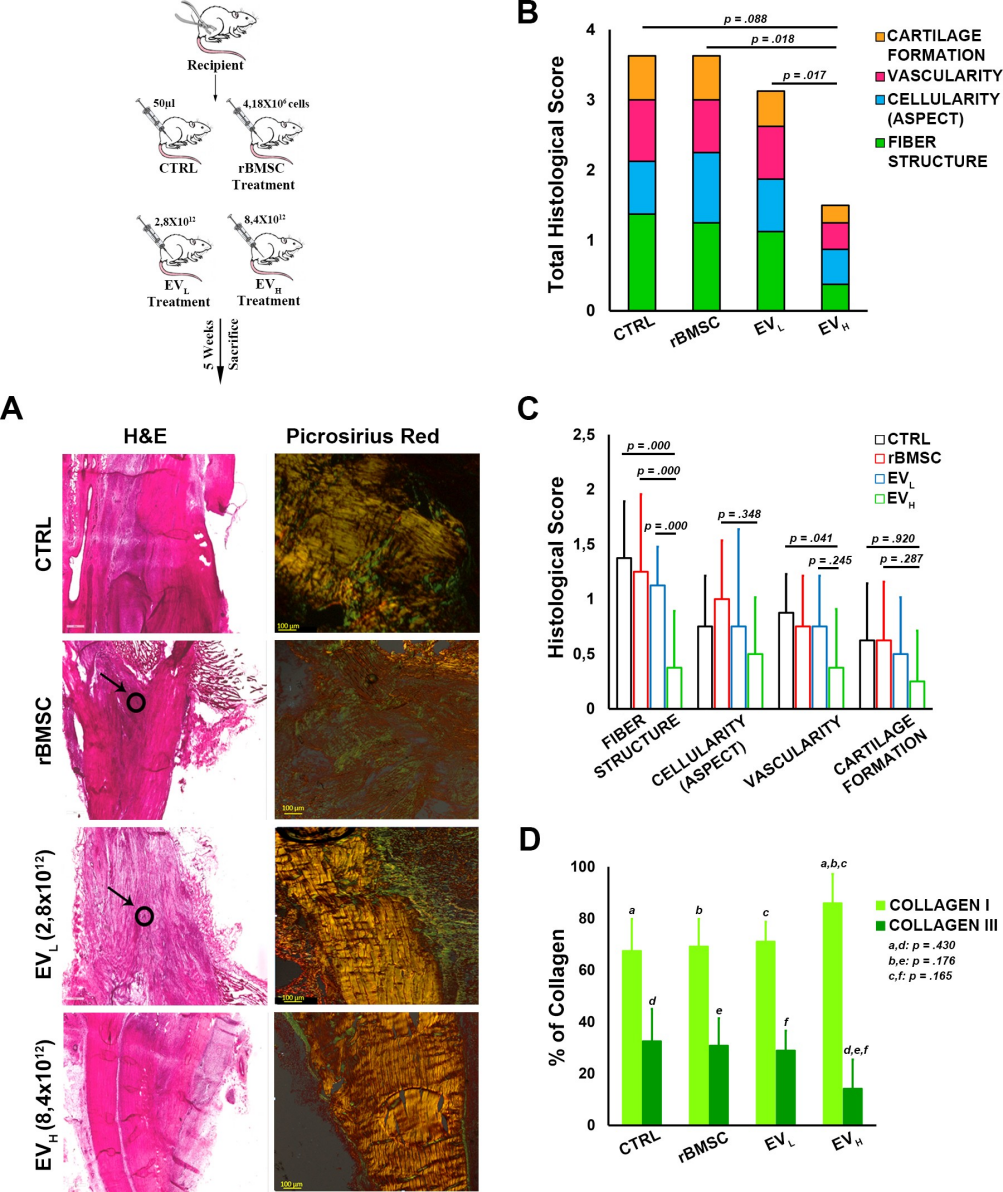
**(A)** Histological analysis of tendon healing at 30 days from injury / start of treatment (on the left panel). The injured Achilles tendons were then stained with H&E for each type of treatment and the quality of tendon repair was evaluated based on properties displayed. [Arrow: (Slight Cartilage Formation)] (x500). On the right panel, longitudinal sections of the right hindlimb rat Achilles tendons stained by Picro-sirius red staining (PSR): (magnification 4×, scale bar = 500 μm). The thicker, mature collagen fibers appear red-orange (type I collagen); the thinner collagen fibers appear pale green (type III collagen). **(B, C)** Stacked histograms of the semiquantitative histomorphometric score for treated and saline control groups. **(B)** Total score for healthy tendon score, including cartilage formation, vascularity, cell appearance, and fiber structure scores (Mean, *n* = 8). **(C)** Detailed sub-scores. **(D)** Histograms of Type I and Type III collagen content, expressed in percentages, for treated, and saline control groups (mean ± SD, *n* = 8). One-way ANOVA test and adjusted Bonferroni (two-way) *post-hoc*. Intra-class correlation for histological scores was excellent for inter-observer reliability (0.79–0.97 depending on score/sub-score). The lesions could be clearly identified on all H&E-stained slices. Histological analysis indicated incomplete restoration of structural integrity.

To investigate whether rBMSC-EVs ameliorate Achilles tendon injury repair *in vivo*, we evaluated their effects in a rat model. Surgical core lesions of the Achilles tendon were produced in both hind limbs of rats, and EVs were immediately injected locally in two different concentrations. In detail, all injured tendons were filled with 50 μL of phosphate-buffered saline (PBS), which contained EV_L_ (2.8x10^12^) and EV_H_ (8.4x10^12^), the same concentration as already used in *in vitro* experiments. As a control, we used a non-treated Achilles tendon injury (i.e. an Achilles tendon injury in which PBS only had been injected) and an rBMSC-treated group (Fig 1). We analyzed the effects of EVs after 30 days on semi-quantitative, histomorphometric results (Fig 5).

The higher concentrations of rBMSC-EVs improve tendon healing, with no detectable local adverse reactions. The total score (Table 1) for the higher concentration (EV_H_) group was significantly lower than for the rBMSC and the EV_L_ (p = .018 and p = .017 respectively) and lower than for the control group (p = .088) (Fig 5B). In greater detail, the sub-scores of the EV_H_ group were lower for fiber structure than other groups, and significantly lower than the rBMSC group (p = .000), EV_L_ (p = .000), and the control group (p = .000); for cellularity scores, the sub-scores of the EV_H_ group were lower than for the rBMSC, the low concentration EV_L_ groups and the control group; for vascularity, the sub-scores of the EV_H_ group were lower than for the rBMSC and low concentration EV_L_ and significantly lower than for the control group (p = .041); the cartilage-formation sub-scores for the higher concentration EV_H_ group were lower than for the rBMSC, low concentration EV_L_ and control groups (Fig 5C). Collectively, in the EV_H_ group, only the cellularity sub-score value was slightly higher than the untreated control group, but we should underline that less cartilage formation, and better fiber structure, were observed than in comparison with all the other treated groups (Fig 5C). In summary, histological evaluation of the EV_H_ group confirmed better restoration of tendon architecture, with optimal alignment of tendon fibers and blood vessels (Fig 5A, 5B and 5C). Analysis of the collagen ratio revealed that the EV_H_ group had a greater effect on tendon-healing than all the other treatments. Specifically, the collagen type I value was higher than in the rBMSC, EV_L_ and control groups; the EV_H_-group collagen-type-III value was lower than in the rBMSC, EV_L_ and control groups (Fig 5D). However, EV_L_ also induced a slight increase in collagen-type-I expression and decrease in collagen-III expression, compared to the rBMSC and control groups. The collagen ratios for the rBMSC group and the EV_L_ group were lower than the untreated saline control value (Fig 5D). It should be noted that the quantity of EVs used in the low concentrations was obtained from the same number of BMSC cells (4 X10^6^) as were used in the tendon injections. In summary, in the tendons treated with a higher concentration of EVs, analysis of the collagen ratios demonstrated higher expression of collagen type I, and lower expression of collagen type III, than in all the other groups, including the untreated saline control group.

The three main phases of tendon healing, featuring distinctive cellular and molecular cascades, are: (i) inflammatory; (ii) reparative or proliferation, characterized by cellularity and matrix production, (mostly collagen type III) and (iii) remodeling (consolidation and maturation), replacing collagen type III with collagen type I and permitting fiber organization[28]. Taken together, results indicate that high concentrations of EVs accelerate tendon healing.

Collectively, the expression of pro-collagen type I and MMP14 led us to hypothesize that, in accordance with studies describing EV-driven, matrix-remodeling in other tissue-types, this may also occur in tendon-tissue repair[29]. Furthermore, a number of studies have shown that MMP-14 plays a pivotal role in collagen homeostasis and remodeling, and, additionally, MMP14 has been demonstrated to potentiate angiogenic processes[30, 31]. The precise role of EV-MMP-14 in tendon healing and repair requires further investigation. We performed experiments *in vitro* on cells isolated from Achilles tendons which were used as recipient cells for rBMSC–EVs: our results very clearly show that EVs at both low and high doses, as used in an *in vivo* model, induce proliferation and migration of tenocyte cells and increase expression of Collagen type I. Our results are in agreement with other recent work by [21], so we may hypothesize, as has already been suggested, that the effects of our EVs vary according to concentration and low and high concentrations probably have distinct roles in overall processes. Furthermore, importantly our experiments demonstrate that EVs induce increased expression and thus production only of Collagen type I and not of collagen type III, which makes them a fundamental part of the repair process for optimal functional repair. Collagen type III is already highly-expressed only in pathological tendons or during the first phase of inflammation/repair. Importantly, the pretreatment with trypsin confirms what others have already shown, namely that lowering the vesicle uptake versus the recipient cells reduces the bioactive effect of the vesicles, and consequently, in our model, the ability to play a role during the tendon repair process. Our animal model study data suggest that EV-treatment accelerates the progression of healing in the remodeling stage of tendon repair in dose-dependent manner. Higher concentrations of MSC-derived EVs performed better than low concentrations and better than MSCs alone. As already shown [9, 10, 11], three studies have already been published on this topic, but direct comparisons with the present investigations are difficult given the different focus. The three previous studies focused largely on inflammatory mechanisms, and used different methodologies and different types of injury over different time periods. Furthermore, variation in EV-application strategies again renders comparisons limited. Most importantly, our study confirms the hypothesis that EVs improve tendon-healing.

In addition, what may be drawn from our study is that the analysis and identification of optimum EV-concentrations over the three-phase tendon-healing process is of fundamental importance and deserves further investigation. High collagen type I expression seems essential to obtain faster tendon healing. The initial increased production of collagen type III shifts to greater collagen type I production early in the healing process. Our data suggest that EV-treatment accelerates the progression of healing in the remodeling stage of tendon repair in a dose-dependent manner, with higher concentrations of EVs influencing the collagen ratio, increasing collagen type I levels. Together with previous studies, it also becomes clear that a delivery support should be used to concentrate treatment at the site of injury. Finally, full understanding of the exact mechanisms through which EVs can influence tendon-healing can only be obtained through extensive study into this most promising approach. A final consideration, in our case, is that only medium-term effects have been evaluated, and further analysis is required to establish the short- and long-term structural and functional benefits, including improved strength and stiffness, of rBMSC-EV treatment in tendon-healing.

Our results enhance knowledge of EVs and their potential for future use as highly effective therapeutic agents in tendon-injury treatments, while avoiding the use of cells, exogenous GFs, or gene delivery systems.

## Supporting information

S1 Fig rBMSC-EVs pre-treated with trypsin abrogate tendon-derived cell proliferation and migration, and increase expression of collagen type I. rBMSC -EVs were pre-treated with trypsin, followed by re-isolation of EVs and application to tendon-derived cells. **A)** Effects of rBMC–EVs on cell proliferation assessed by MTT assay at 48 hr, with different concentration of rBMC–EVs (2,8x10^12^ or 8,4x10^12^ /mL), with or without trypsinization. **B)** tenocyte migration assessed by scratch wound healing assay at 24 hr with rBMSC -EVs (2,8x10^12^ or 8,4x10^12^ /ml), with or without trypsinization. **C**) Expression of collagen type I after 7 d of rat tenocyte culture with rBMSC -EVs (2,8x10^12^ or 8,4x10^12^ /ml), with or without trypsinization. The expression of collagen type I was assessed by anti-collagen I-alexa-fluor 488 staining. The mean fluorescent intensity/pixel was measured and expressed to corresponding tendon-derived cell. Collagen type I Intensity (Total Area was quantified by anti-collagen type I) was measured by Nikon software. Data shown as mean ± SD, and represent triplicate experimental replicates. *p<0.05; **** *p* < 0.00001.
(TIF)

S1 Raw images Berardi et al. (PDF)

## Abbreviations

MSCs: Mesenchymal stromal/stem cells
EVs: extracellular vesicles
rBMSCs-EVs: rat-bone-marrow-MSC-derived EVs
ECM: extracellular matrix
MMP14: matrix metalloproteinases 14
BMSCs: bone marrow-derived mesenchymal stromal/stem cells
MISEV: International Society for Extracellular Vesicles
AFM: Atomic Force Microscopy
CONAN: Colloidal nanoplasmonic
PBS: phosphate-buffered saline
L: Low
H: High
