# Supplementary figures and images for: Extracellular vesicles from rat-bone-marrow mesenchymal stromal/stem cells improve tendon repair in rat Achilles tendon injury model in dose-dependent manner: A pilot study
Source: PLoS One. 2020 Mar 12;15(3):e0229914. doi: 10.1371/journal.pone.0229914 (PMC7067391; doi:10.1371/journal.pone.0229914)

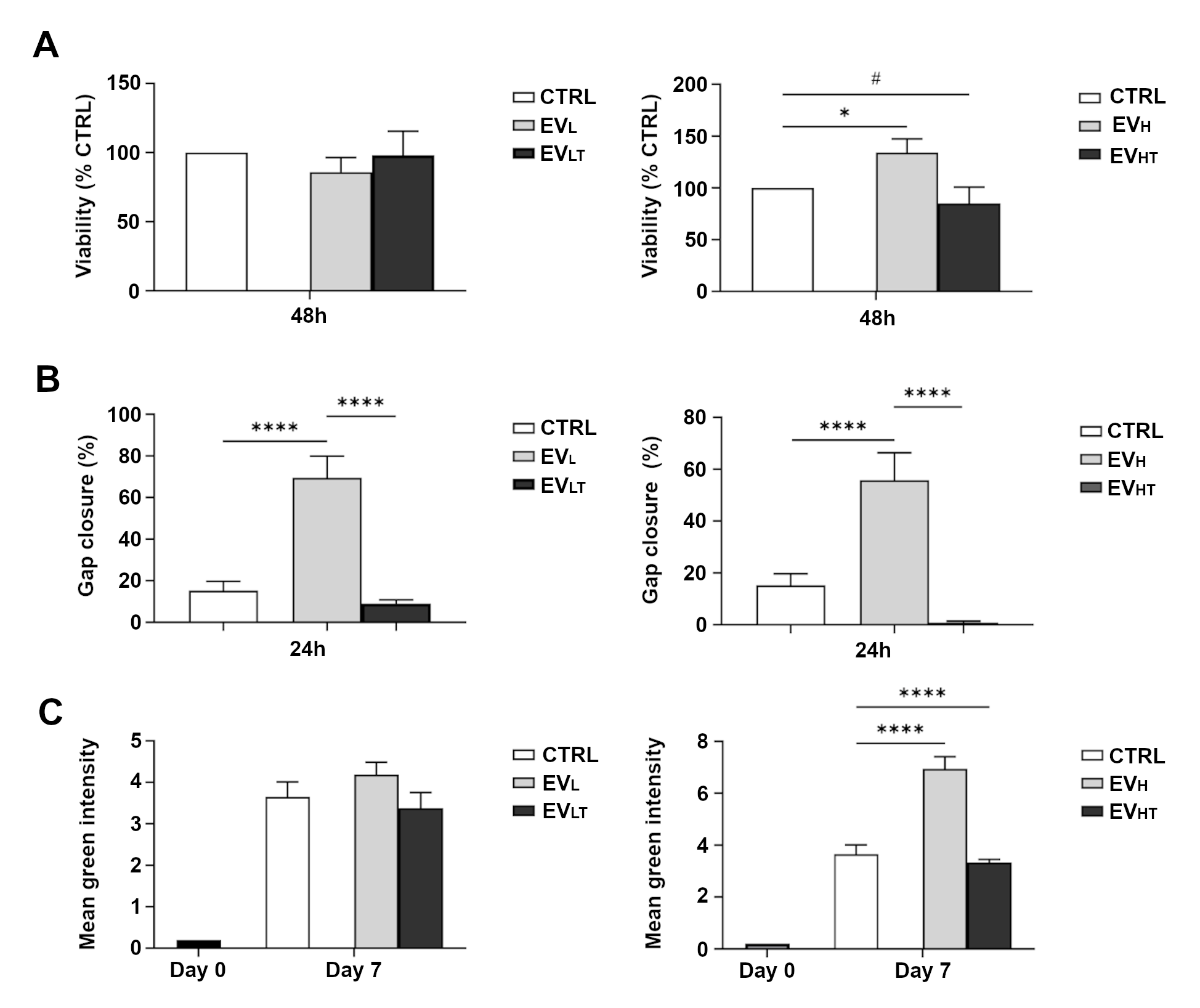

Supplement: S1 Fig — rBMSC -EVs were pre-treated with trypsin, followed by re-isolation of EVs and application to tendon-derived cells. A) Effects of rBMC–EVs on cell proliferation assessed by MTT assay at 48 hr, with different concentration of rBMC–EVs (2,8x10^12^ or 8,4x10^12^ /mL), with or without trypsinization. B) tenocyte migration assessed by scratch wound healing assay at 24 hr with rBMSC -EVs (2,8x10^12^ or 8,4x10^12^ /ml), with or without trypsinization. C) Expression of collagen type I after 7 d of rat tenocyte culture with rBMSC -EVs (2,8x10^12^ or 8,4x10^12^ /ml), with or without trypsinization. The expression of collagen type I was assessed by anti-collagen I-alexa-fluor 488 staining. The mean fluorescent intensity/pixel was measured and expressed to corresponding tendon-derived cell. Collagen type I Intensity (Total Area was quantified by anti-collagen type I) was measured by Nikon software. Data shown as mean ± SD, and represent triplicate experimental replicates. *p<0.05; **** p < 0.00001. (TIF) [file pone.0229914.s001.tif]

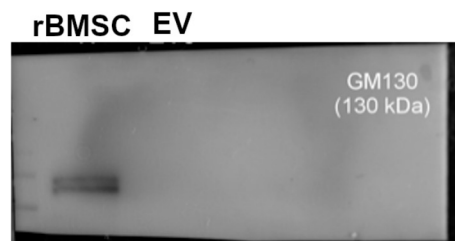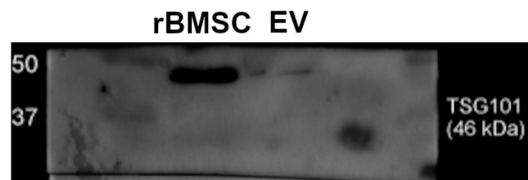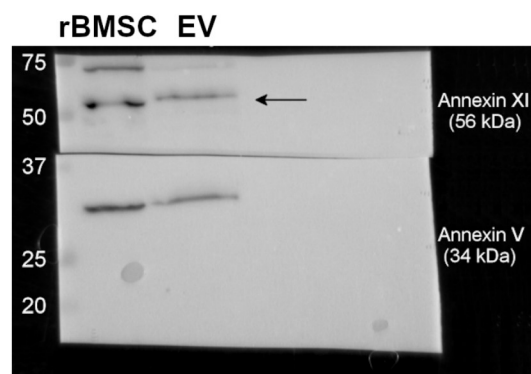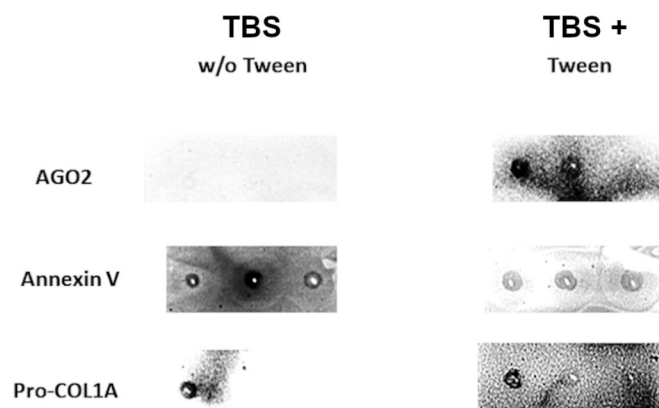

**EV**

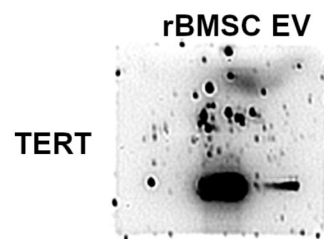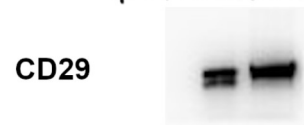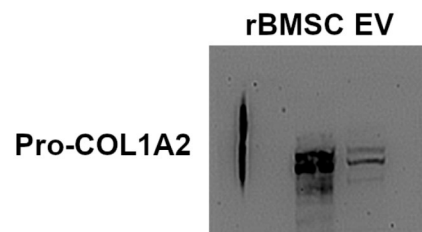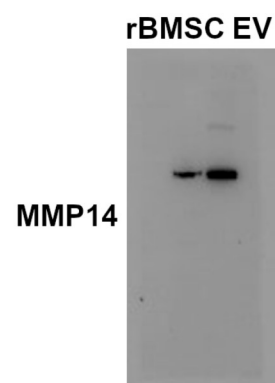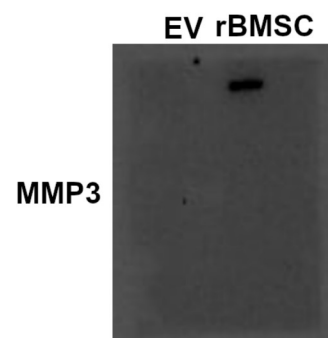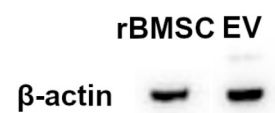

Supplement: S1 Raw images — (PDF) [file pone.0229914.s002.pdf]
